# Supplementary material for: Adult responses to infant prelinguistic vocalizations are associated with infant vocabulary: A home observation study
Source: PLoS One. 2020 Nov 25;15(11):e0242232. doi: 10.1371/journal.pone.0242232 (PMC7688127; doi:10.1371/journal.pone.0242232)
Supplement: Attachment — Submitted filename: Response to Reviewers.docx [file pone.0242232.s001.docx]

UCM

UNIVERSITY OF CALIFORNIA, MERCED

BERKELEY	∙ DAVIS	∙	IRVINE	∙	LOS ANGELES	∙	RIVERSIDE	∙	SAN DIEGO	∙	SAN FRANCISCO

SANTA BARBARA	∙	SANTA CRUZ

PSYCHOLOGICAL SCIENCES

5200 N. LAKE ROAD

MERCED, CALIFORNIA 95343

9/15/2020

Dear Editor Nomikou,

We submit for your consideration a revision of our manuscript, titled “Adult responses to infant prelinguistic vocalizations are associated with infant vocabulary: A home observation study,” submitted as a Research Article to *PLOS ONE.* In what follows, our responses to each point are detailed in *italics*, with the reviewers’ comments given in quotes.

Thank you once again for your thoughtful consideration of our work, and for the opportunity to improve it. We feel that the paper has been improved with the benefit of the feedback that we have received and look forward to your assessment.

Sincerely,

Lukas Lopez (on behalf of all co-authors)

Department of Psychological Sciences

University of California, Merced

Merced, CA 95343, USA

Email: [llopez65@ucmerced.edu](mailto:llopez65@ucmerced.edu)

**Replies to Reviewer:**

**Reviewer #1:**

“First, I think the framing and motivation of the paper isn’t strong enough and could use more nuance. The study is motivated by the naturalistic method, suggestions that such method is more ecologically valid than the structured lab environment. I wholeheartedly agree. There aren’t enough studies focused on what parents actually do in the confines of their homes and how that shifts across the day. However, it isn’t that lab studies don’t generalize at all or capture something completely artificial and different from naturalistic environments. Lab studies, rather, capture a specific aspect of interactions that is found in the home environment. Tamis-LeMonda and colleagues’ paper (citation 16 in the manuscript) on methods shows that structured observations (which were done in the parents home, but are similar to structured observations in lab environments) capture the highest consecutive five minutes of interaction in the home. They capture the chattiest moments, that is. It is no surprise that then years of studies on language input during structured observations predict child language development. it isn’t the the lab methods are invalid, they do capture something meaningful. They just don’t capture ecology, fluctuation of talk across the day, the setting and activity within which interactions take place and learning occurs, the most interesting and embodied aspects of learning. The aspects of children’s context that are largely ignored by developmental psychologists who focus on language but are constantly articulated by cultural psychologists and linguistic anthropologists are necessary. This point I think is important to address in the introduction, and necessitates a stronger motivation for the study to be articulated by the authors.”

*We thank the reviewer for the positive assessment of the methodologies used in the present paper and the excellent suggestion to further highlight the behavioral ecology of the observed behaviors. We agree that while in-lab observations capture meaningful caregiver behaviors, studies in the naturalistic home environment capture the ecology in which these behaviors normally transpire, including a much greater range of settings and activities such as cultural contexts and caregiving practices. We now reference work from cultural psychology and anthropology that highlight these points and have emphasized the utility of naturalistic recordings for capturing a variety of learning ecologies in the Introduction of the revised manuscript (p. 4, lines 66-69; p. 7, lines 137-142; & p. 8, lines 160-163).*

“Second, information presented about contingent responsiveness is incomplete. It seems that the authors captured base rates of responses, that is, frequencies of how many times parents responded to the different types of vocalizations. In work on contingent responsiveness, typically researchers would go a step further and conduct sequential analyses to gather probabilities of mother responding to infant vocalizations. Such probabilities account for base rates of behaviors, and can be used in analyses predicting language outcomes. These probabilities matter because they differentiate mothers who respond to an infant twice (but infants’ total vocalizations are 10) from a mother who responds to an infant twice (but infants’ total vocalizations are 3). The second mother is more responsive than the first. On line 305 the authors state that they are not seeking to detect causal effects of infants’ vocalizations, which is a slight mischaracterization of the baseman’s GSEQ method. They are probabilities, and they take into account all of the mother and infant talk. On page 29, the authors use the language “elicit” which appears causal anyhow. So my questions are: if authors are trying to extend prior literature into the home and see if patterns found in structured observations in lab (or home!) mirror, why not use established methods for contingent responsiveness?”

*We thank the reviewer for this suggestion and for pointing out the places where we have inadvertently somewhat misrepresented the prior literature and been sloppy in our use of terminology implying causal relationships!*

*We agree that matching previous methodologies will indeed increase the generalizability about how behaviors observed in the lab translate to the naturalistic home environment. Past studies relating parent-infant interactions in more constrained settings to infant vocabulary and language outcomes have used a variety of different methodologies. Sometimes the focus has been on controlling for base rates and other times it has not, and when it has controlled for base rates, the methods used have varied. The models in the present paper controlled for the overall number of infant vocalizations and caregiver infant-directed utterances in Step 1 and for the overall number of infant canonical vocalizations and caregiver responses in Step 2, thus controlling for the base rates of infants’ vocalizations and caregivers’ responsiveness. We have added additional text in the revised manuscript to clarify how we controlled for the base rates of these vocal behaviors (p. 19, lines 409-410).*

*We have edited the text in the Sequence Codes subsection of the Method section so that it no longer implies that sequential analyses (such as GSEQ) intend to detect causal relationships. We have also replaced the first instance of “elicit” in the Discussion section from the prior submission with a statement that implies less causality. We did, however, keep the term when discussing the potential feedback loop that we believe the results support—but we added mention that that feedback loop is “theoretical” (p. 30, lines 630-635).*

“Regardless, the authors should then also report how many infant vocalizations occurred in each segment that the mothers did not respond to to give readers a sense of the overall interaction. In the context of the home, unlike the structured context, mothers may attend to other tasks, and interactions may be fluid, comprised of smaller bouts perhaps. I would presume that mothers would have lower rates of responding to total number of infant vocalizations in the home than in the lab, where they have little else to do but interact, and my hypothesis would also be that bouts of very contingent interactions would focus on specific activities, like book sharing or play.”

*We agree with the reviewer that this would be useful descriptive information to present in the paper. We have now included the average rate in which caregivers contingently responded to infant speech-related vocalizations in the observed segments (p. 20, lines 435-436).*

*Additionally, controlling for caregiver response rates in Step 1 in all of the models does not change the results. We have added this information in text (p. 18, lines 392) and have added the output for the models controlling for caregiver response rates to the SOM (see S12 & S13).*

*Furthermore, we have added the number of infant vocalizations that did and did not receive contingent caregiver responses for each infant to the updated dataset on OSF. Additionally, we added a ZIP file that contains all of the timestamped excel exports from which these data were derived. Specifically, each export contains timestamped infant vocalization and caregiver response codes for each participant. This way researchers interested in probing these contingencies further have all of the data at their disposal to do so.*

“Finally, I appreciate that the authors noted the types of activities that occurred in the segments chosen, and I think that those should be highlighted. The study focuses on language interactions in the context of everyday life, and so interpretation of input in the context of play specifically (which comprised most segments it appears) is meaningful and necessary. It should also then be linked to prior work on language input by activity context, perhaps in the discussion.”

*The reviewer raises an excellent point that the contexts in which the interactions took place may have important implications of our findings. We have added further discussion of this point in the Future Directions section of the Discussion (p. 31, lines 666-670).*

**Reviewer #2:**

“This is a well written manuscript that details the findings for in home parent-infant vocal interactions and their relation to language outcomes. This is an area of research that is underrepresented in the literature, as the majority of studies have been conducted in the lab. The meticulous coding captures specific responses (recasts, expansions) to more advanced infant vocalizations (canonical vocalizations) that support language development. The findings reveal important moment to moment vocal exchanges that can support positive outcomes. I appreciate the limitations discussed by the authors, but I would also suggest that there are potential applications of their research that they could elude to in the discussion. I agree that these are just short snippets of exchanges, but the importance of uncovering which specific vocalizations and feedback (loops) is significant for understanding how parents support language in day to day interactions.”

*We thank the reviewer for noting the importance of the current findings. The revised manuscript now includes additional potential applications of how the present results may inform caregiver practices that facilitate positive language outcomes in the home environment in the Discussion (p. 28, lines 586-588 & ps. 30-31, lines 642-656).*
